# Ideal cardiovascular health status and risk of cardiovascular disease and all-cause mortality: over a decade of follow-up in the Tehran lipid and glucose study

**DOI:** 10.3389/fcvm.2022.898681

**Published:** 2022-08-04

**Authors:** Farzad Hadaegh, Somayeh Hosseinpour-Niazi, Niloofar Deravi, Mitra Hasheminia, Nazanin Moslehi, Hossein Toreyhi, Fereidoun Azizi

**Affiliations:** ^1^Prevention of Metabolic Disorders Research Center, Research Institute for Endocrine Sciences, Shahid Beheshti University of Medical Sciences, Tehran, Iran; ^2^Nutrition and Endocrine Research Center, Research Institute for Endocrine Sciences, Shahid Beheshti University of Medical Sciences, Tehran, Iran; ^3^Endocrine Research Center, Research, Institute for Endocrine Sciences, Shahid Beheshti University of Medical Sciences, Tehran, Iran

**Keywords:** ideal cardiovascular health, cardiovascular disease, all-cause mortality, cohort study, Iranian

## Abstract

**Objective:**

To quantify the association between ideal cardiovascular health (CVH) metrics and incident cardiovascular disease (CVD) including different subtypes [coronary heart disease (CHD), stroke, and sudden death], and all-cause mortality in an Iranian population.

**Methods:**

The study population included 6,388 participants (2,726 men) aged 48.0 ± 12.4 years free of CVD at baseline. We utilized the American Heart Association’s 2020 impact target criteria of ideal, intermediate, and poor CVH. The multivariate Cox proportional Hazard model, adjusted for age, sex, educational level, marital status, and family history of CVD, was applied to estimate the hazard ratio (HR) of outcomes per one additional metric of ideal CVH metrics. Furthermore, the risk was also calculated for ideal and intermediate categories considering poor category as a reference.

**Results:**

During the median follow-up of 11.26 years, 692 CVD, 589 CHD, 130 stroke, 111 sudden death, and 519 all-cause mortality events were reported. All of the individual ideal CVH metrics were independent predictors except intermediate physical activity level for CVD, BMI < 25 kg/m^2^, and intermediate physical activity for all-cause mortality. Each additional metrics of ideal CVH decreased the risk by 31 (0.69, 0.65–0.73) for CVD, 32 (0.68, 0.64–0.73) for CHD, 31 (0.69, 0.60–0.80) for stroke, 25 (0.75, 0.64–0.88) for sudden death, and 13% (0.87, 0.81–0.93) for all-cause mortality events. Moreover, intermediate and ideal categories of CVH metrics were associated with lower risk for different CVD outcomes, i.e., 44 (0.56, 0.48–0.65) and 76% (0.24, 0.17–0.35) for CVD; 43 (0.57, 0.47–0.67) and 75% (0.25, 0.16–0.37) for CHD, 58 (0.42, 0.29–0.61) and 86% (0.14, 0.04–0.44) for stroke; 56 (0.44, 0.29–0.66) and 55% (0.45, 0.21–0.99) for sudden death; and 25 (0.75, 0.62–0.90) and 46% (0.54, 0.37–0.80) for all-cause mortality events, respectively. We also assessed the impact of changes in ideal CVH status from phase III to phase IV (2008–2011) on CVD events among 5,666 participants. Accordingly, compared to those remaining in the poor category, all of the changes in ideal CVH categories showed a lower risk for CVD events.

**Conclusion:**

Among the Iranian population, meeting higher ideal CVH metrics is associated with a lower risk of different CVD events and mortality outcomes.

## Introduction

Cardiovascular disease (CVD) is responsible for the greatest proportion of premature non-communicable chronic disease (NCD) mortality globally (1). Increasing evidence has suggested that shared lifestyle and biological risk factors, such as unhealthy diet, hypertension, physical inactivity, dyslipidemia, and obesity may increase incident CVD risk (2, 3). As a strategy for reducing CVD risk and overall premature NCD mortality, the World Health Organization (WHO) has given special emphasis to reducing these risk factors (4).

The American Heart Association (AHA) established a simplified 7-item tool in 2010 to promote ideal cardiovascular health (CVH), encompassing physical activity, smoking status, healthy dietary intake, total cholesterol, body mass index (BMI), fasting plasma glucose (FPG), and blood pressure (5). Since then, the concept of ideal cardiovascular health (CVH) has been widely used to evaluate population health (6).

A recent meta-analysis of 12 prospective studies reported that compared with high-risk individuals (adults who met 0–2 of the ideal CVH metrics), a 47% lower risk for CVD incidence was observed in those who had 3–4 points for the ideal CVH metrics and also a 72% lower risk in those with 5–7 points (7). It should be noted that significant heterogeneities (I^2^ ranged from 70.5 to 71.2%) across earlier research were reported in the aforementioned meta-analysis. Moreover, the included studies were mainly conducted among the United States, European, and Chinese populations. Additionally, studies in the general population, which were performed after the above-mentioned meta-analysis, were mainly conducted in East Asia (8–12). To the best of our knowledge, no study has yet evaluated the association between ideal CVH and incident CVD in the Middle East and North Africa (MENA) region with the high burden of CVD risk factors (13). Recently, using three cross-sectional STEP-wise approaches to surveillance (STEPS), we found that in 2016 compared to 2007, the prevalence of hypertension, hypercholesterolemia, current smoking, and normoglycemia declined, whereas poor physical activity level, poor healthy diet score, and being overweight/obesity increased. Generally, despite some improvements in CVH metrics, less than 4% of the Iranian population met ≥ 6 ideal CVH metrics in 2016 (14).

In this study, we aimed to quantify the association between ideal CVH metrics and incident CVD and its subtypes, including coronary heart disease (CHD), stroke and sudden death, and all-cause mortality in a population-based cohort called the Tehran and Lipid Glucose Study (TLGS) during more than a decade of follow-up.

## Materials and methods

### Study design and population

The design and registration of TLGS have been previously detailed (15). The TLGS is a longitudinal survey performed on people aged ≥ 3 years who live in Tehran’s metropolitan region. The purpose of the cohort is to ascertain the prevalence and incidence of non-communicable diseases, as well as their related outcomes. The TLGS was enrolled in two stages: from 1999 to 2001 (*n* = 15,005) and from 2001 to 2005 (*n* = 3,550). Information was gathered about every 3 years in face-to-face visits by the local research team to update the previous data.

For the current study, we included 7,631 participants aged 30–80 years from phase III TLGS conducted in 2005–2008. From this number, we excluded those with prevalent CVD including cases with self-reported stroke, acute myocardial infarction, acute coronary syndrome, or having percutaneous coronary intervention or coronary artery bypass graft (*n* = 749) and missing data on covariates including age, sex, educational level, marital status, BMI, smoking, physical activity, blood pressure, serum cholesterol, diabetes status (*n* = 460, considering overlap features between missing covariates), and those without any follow-up (*n* = 34), leading to 6,388 (2,726 men), who were followed up until 20th March 2018 (Supplementary Figure 1). To examine the impact of ideal CVH on all-cause mortality, we did not exclude cases of prevalent CVD, so the data analysis was performed among 7,036 (3,972 men).

Due to the inadequate dietary data for all participants, our primary analysis in this research was based on six criteria of ideal CVH. Dietary data were added to a subsample with nutritional information (*N* = 1,837) for sensitivity analysis. Of those, 82 were excluded because their daily energy intakes were < 500 or > 4,200 kcal. Finally, 1,755 participants remained to compute the ideal CVH based on all seven components.

We entered the participants in phase III of TLGS for data analysis because dietary assessment in phases I and II of the TLGS was gathered using 24-h recall, and this method is not an accurate tool to assess the dietary intake (16). In addition, the number of individuals with dietary data in phases I and II was too small (*n* = 819).

The research committee of the Research Institute for Endocrine Sciences at Shahid Beheshti University of Medical Sciences authorized the study concept, and each subject provided written informed permission.

### Clinical and laboratory measurements

A standard questionnaire was used to collect information on demographics, age, educational level, physical activity, smoking status, marital status, medication, and family history of CVD (15). A validated semi-quantitative food frequency questionnaire was administered to determine the frequency of consumption of each food item on a daily, weekly, or monthly basis in the past year (17). To assess participants’ physical activity, we used the Persian-translated version of the Modifiable Activity Questionnaire (MAQ). The MAQ defined physical activity in terms of average metabolic equivalent tasks (METs) rather than duration of physical activity (as in the AHA’s 2020 impact targets) (18).

Participants’ height and weight were measured after they removed their shoes and wore light clothing. In a standing position with the shoulders in normal alignment, height was measured using a tape meter. Weights were taken to the closest 0.1 kg. The BMI (kg/m^2^) was calculated by dividing weight (kg) by height (m) squared.

Two systolic blood pressure (SBP) and diastolic BP (DBP) measurements were taken on the right arm following a 15-min sitting rest period in accordance with the TLGS design (19). The mean of two measurements was used to calculate the subject’s BP. After a 12–14 h overnight fast, all research participants had their blood collected between 7:00 and 9:00 a.m. Laboratory measurements have been thoroughly discussed elsewhere (19). All blood tests were done at the TLGS research laboratory on the day of blood collection. Serum total cholesterol and FPG were measured using an enzymatic colorimetric method with cholesterol oxidase and cholesterol esterase, and glucose oxidase enzymes, respectively.

### Ideal cardiovascular health criteria

We utilized the American Heart Association’s 2020 impact target criteria of ideal, intermediate, and poor CVH (5). According to AHA definitions, the following seven ideal CVH metrics, which include behavioral and biological aspects, were graded as ideal, intermediate, or poor. Behavioral factors include: (1) smoking status: ideal (never or quit for more than 12 months), intermediate (former smoker or quit for ≤ 12 months), and poor (current smoker); (2) BMI: ideal (< 25 kg/m^2^), intermediate (25–29.9 kg/m^2^), and poor ≥ 30 (kg/m^2^); (3) physical activity: ideal (≥ 1,500 METs min/week), intermediate (600–1,500 METs min/week), or poor (< 600 METs min/week); (4) diet (in accordance with the AHA) was classified into five components: at least 4.5 cups/day of fruits and vegetables; at least two 3.5-ounce servings/week of fish; < 1,500 mg/day of sodium; ≤ 36 ounces/week of sugar sweetened beverages (SSBs); and at least three 1-ounce servings/day of whole grains: ideal (four to five components), intermediate (two to three components), and poor (0 to one component). Biological factors include: (1) BP (SBP/DPB): ideal (< 120/80 mmHg if untreated), intermediate (120–139/80–89 mmHg or treated), and poor (≥ 140/90 mmHg); (2) total cholesterol: ideal (< 200 mg/dL if untreated), intermediate (200–239 mg/dL or treated), and poor (> 240 mg/dL); and (3) FPG: ideal (< 100 mg/dL if untreated) intermediate (100–125 mg/dL or treated), and poor (≥ 126 mg/dL). Ideal, intermediate, and poor ideal CVH metrics are defined as the presence of ≥ 5, 3–4, and ≤ 2 ideal CVH metrics, respectively (5).

To assess the behavioral (physical activity, smoking status, BMI, and diet), biological (total cholesterol, FPG, and BP), and global ideal CVH (both behavioral and biological CVH), 0 (poor or intermediate) or 1 (ideal) point were allocated to each metrics of CVH. Ideal CVH was defined as ideal, ≥ 5; intermediate, 3–4; and poor, 0–2.

### Definition of terms

Type 2 diabetes (T2DM) was defined as FPG ≥ 7 mmol/L, or using anti-diabetic medication based on the American Diabetes Association (20). History of CHD or stroke prior to the age of 55 years in male relatives and prior to the age of 65 years in female relatives was regarded as a family history of CVD.

### Outcomes

As previously reported, all TLGS participants are contacted annually for any medical incidents that result in hospitalization throughout the previous year (19). A skilled nurse inquired about any medical problems, and then an expert physician gathered further information about the case *via* a home visit and data gathering from medical records. The death certificate, forensic medicine report, and verbal autopsy were obtained in death cases. Internists, endocrinologists, cardiologists, epidemiologists, and other experts, if required, assessed the acquired data to provide a result for a case by an outcome committee. The TLGS outcome committee scrutinized and assessed each fatal case in great detail.

CVD, and its subtypes (CHD, stroke, and sudden death) and all-cause mortality were considered outcomes. Any CHD events and fatal and non-fatal strokes were included in the definition of CVD. CHD was classified as definite MI (diagnostic electrocardiography and biomarkers), probable MI (positive electrocardiograph findings plus cardiac symptoms or signs plus missing biomarkers or positive electrocardiograph findings plus equivocal biomarkers), and angiographic proven CHD. Incident stroke was defined as all cases of definite and possible stroke and transient ischemic attack (21). “Definite sudden death” was defined as a sudden pulseless state related to the cardiac source in a previously stable individual. Possible sudden death was known as unpredictable death 24 h after last having been observed alive that was not related to a specific origin of circulatory collapse or an underlying source other than the heart. In the current study, definite and possible sudden deaths were defined as sudden death (22). Death from any cause was classified as “all-cause mortality.” In the TLGS, outcomes are coded according to ICD-10 criteria and AHA classification for cardiovascular events [i.e., ischemic heart disease (ICD10 codes I20-I25), sudden cardiac death (I46.1), or stroke (ICD-10 codes I60-I69)] (23).

### Statistical analysis

The baseline characteristics are presented as mean ± SD for variables with normal distribution, median (interquartile range) for those with skewed distribution, and count (percentage) for dichotomous variables. To compare the baseline characteristics across ideal CVH categories, the analysis of variance (ANOVA) test and Pearson’s chi-squared test were applied as appropriate.

Multivariate adjusted hazard ratios (HRs) were calculated for each of the ideal CVH metrics, considering the poor status as the reference in both model 1 (adjusted for age and sex) and model 2 (further adjusted for educational level, marital status, and family history of CVD). The Cox Proportional Hazard (PH) model was also used to calculate the HRs of CVD and all cause-mortality for ideal and intermediate categories, considering the poor category as a reference, in models 1 and 2. For all-cause mortality as the outcome, we also adjusted model 2 for prevalent CVD. The Cox PH model was also used to estimate the HRs of the outcome of interest per one additional ideal CVH metric as well as for combined ideal CVH metrics.

Since we did not find any interaction between sex and ideal CVH categories in multivariate analysis for CVD and its subtypes (all *P*-values > 0.5) and all-cause mortality (all *P*-values > 0.1), the analysis was performed in the pooled sample to reach full statistical power. Statistical tests based on the scaled Schoenfeld residuals and the log–log plots were used to validate the PH assumptions in the Cox models and no violation was found. Statistical analyses were performed in SPSS version 20.0 (SPSS Inc., Chicago, IL, United States) and Stata version 14.0 (StataCorp LLC, TX, United States).

## Results

Baseline characteristics of the study population by ideal CVH metrics are shown in Table 1. Among a total of 6,388 participants (men: 2,726), the number (%) of ideal, intermediate, and poor CVH was 1,827 (28.6%), 3,410 (53.4%), and 1,151 (18.0%) respectively. The mean age and BMI of the study population were 48.0 ± 12.4 years and 28.2 ± 4.6 kg/m^2^, respectively. There were significant differences between categories of ideal CVH for all of the baseline characteristics, except for family history of CVD.

**TABLE 1:** Baseline characteristics by global cardiovascular health status, Tehran Lipid and Glucose Study (TLGS), 2005–2018.

	Global Cardiovascular Health Status*				
	Overall (*n* = 6,388)	Poor (*n* = 1,827)	Intermediate (*n* = 3,410)	Ideal (*n* = 1,151)	*P*-value
Continuous variables, Mean ± SD					
Age (year)	48.0 ± 12.4	53.0 ± 12.0	47.6 ± 12.0	41.3 ± 10.5	**<0.001**
BMI (kg/m^2^)	28.2 ± 4.6	30.2 ± 4.5	28.2 ± 4.3	25.0 ± 3.9	**<0.001**
SBP (mmHg)	116.5 ± 18.7	128.2 ± 18.2	114.6 ± 17.2	103.5 ± 11.8	**<0.001**
DBP (mmHg)	74.8 ± 10.3	80.4 ± 9.9	74.1 ± 9.7	67.8 ± 7.7	**<0.001**
FPG (mg/dL)	90.0 (84.0–99.0)	101.0 (90.0–118.0)	89.0 (84.0–95.0)	86 (81.0–90.0)	**<0.001**
Total cholesterol (mg/dL)	196.0 ± 38.6	217.3 ± 37.8	193.4 ± 36.4	170.1 ± 25.9	**<0.001**
HDL cholesterol (mg/dL)	41.5 ± 10.2	40.4 ± 9.8	41.2 ± 10.1	44.1 ± 10.5	**<0.001**
Categorical variables, number (%)					
Sex (male)	2,726 (42.7)	918 (50.2)	1,453 (42.6)	355 (30.8)	**<0.001**
Educational level (year)					
≤ 6	1,967 (30.8)	794 (43.5)	1,005 (29.5)	168 (14.6)	**<0.001**
6–12	3,316 (51.9)	788 (43.1)	1,814 (53.2)	714 (62.0)	**<0.001**
> 12	1,105 (17.3)	245 (13.4)	591 (17.3)	269 (23.4)	**<0.001**
Low physical activity	2,239 (35.1)	935 (51.2)	1,142 (33.5)	162 (14.1)	**<0.001**
Current smoking	787 (12.3)	342 (18.7)	398 (11.7)	47 (4.1)	**<0.001**
Marital status					
Married	5,571 (87.2)	1,538 (84.2)	3,010 (88.3)	1,023 (88.9)	**<0.001**
Widowed + Divorced	535 (8.4)	242 (13.2)	243 (7.1)	50 (4.3)	**<0.001**
Single	282 (4.4)	47 (2.6)	157 (4.6)	78 (6.8)	**<0.001**
Glucose lowering drug use, yes	394 (6.2)	276 (15.1)	112 (3.3)	6 (0.5)	**<0.001**
Anti-hypertensive drug use, yes	312 (4.9)	191 (10.5)	114 (3.3)	7 (0.6)	**<0.001**
Lipid-lowering drug use, yes	299 (4.7)	181 (9.9)	116 (3.4)	2 (0.2)	**<0.001**
Family history of CVD, yes	1,413 (22.1)	424 (23.2)	733 (21.5)	256 (22.2)	0.361
T2DM	637 (10%)	451 (24.7)	179 (5.2)	7 (0.6)	**<0.001**

Values are mean ± SD, median (interquartile range) or n (%). *Defined according to the number of ideal metrics: 0–2 (poor), 3–4 (intermediate), and 5–6 (ideal). CVH, Cardiovascular health; BMI, Body mass index; SBP, Systolic blood pressure; DBP, diastolic blood pressure; FPG, Fasting plasma glucose; CVD, Cardiovascular disease; T2DM, Type 2 diabetes mellitus. The statistically significant data is bold.

During the median (interquartile range) follow-up of 11.26 years (10.67–12.35), 692 CVD events (men = 389) were reported, of which 31, 305, and 356 events occurred among individuals with ideal, intermediate, and, poor global CVH, respectively. Also, among 7,035 individuals, 519 deaths (CVD 40.2%, cancer 20.6%, diabetic complications 3.3%, accidents 3.5%, infection 13.3%, and miscellaneous 19.1%) were documented.

The HRs (95% CI) of the intermediate and ideal statuses of each CVH metric, for CVD and all-cause mortality events, are shown in Table 2. Intermediate status of BMI, total cholesterol, BP, and FPG was associated with a decreased risk of 22 (0.66–0.93), 26 (0.60–0.90), 28 (0.40–0.86), and 44% (0.45–0.71) for incident CVD after adjustment for confounding factors (model 2). Moreover, the corresponding values for the ideal status of the mentioned CVH metrics were 44 (0.45–0.70), 50 (0.41–0.61), 66 (0.36–0.54), and 60% (0.33–0.48). The ideal status of smoking and physical activity were also associated with a decreased risk of 44 (0.53–0.83) and 22% (0.65–0.92), respectively.

**TABLE 2:** The risk of intermediate and ideal status for each metrics for cardiovascular disease and all-cause mortality: Tehran Lipid and Glucose Study (TLGS), 2005–2018.

	CVD					All-cause mortality				
	n/N	Model 1		Model 2		n/N	Model 1		Model 2	
		HR (95%CI)	*p*-value	HR (95%CI)	*p*-value		HR (95%CI)	*p*-value	HR (95%CI)	*p*-value
Smoking status	692/6,388					519/7,036				
- Poor	105/787	1.00		1.00		492/5,057	1.00		1.00	
- Intermediate	95/544	0.85 (0.64–1.13)	0.258	0.88 (0.67–1.17)	0.389	85/653	**0.70 (0.51–0.96)**	**0.028**	**0.69 (0.50–0.95)**	**0.024**
- Ideal	492/5,057	**0.64 (0.51–0.80)**	**<0.001**	**0.66 (0.53–0.83)**	**<0.001**	363/5,521	**0.52 (0.39–0.68)**	**<0.001**	**0.54 (0.41–0.71)**	**<0.001**
Body mass index	692/6,388					519/7,036				
- Poor	246/1,948	1.00		1.00		159/2,166	1.00		1.00	
- Intermediate	314/2,897	**0.76 (0.64–0.90)**	**0.002**	**0.78 (0.66–0.93)**	**0.006**	217/3,196	0.78 (0.63–0.96)	0.020	**0.80 (0.65–0.98)**	**0.035**
- Ideal	132/1,543	**0.54 (0.43–0.67)**	**<0.001**	**0.56 (0.45–0.70)**	**<0.001**	143/1,674	**0.86 (0.67–1.09)**	**0.213**	0.92 (0.72–1.17)	0.491
Physical activity	692/6,388					519/7,036				
- Poor	266/2,239	1.00		1.00		215/2,488	1.00		1.00	
- Intermediate	158/1,430	0.91 (0.75–1.11)	0.349	0.91 (0.75–1.11)	0.374	120/1,579	0.81 (0.65–1.02)	0.073	0.84 (0.67–1.05)	0.131
- Ideal	268/2,719	**0.79 (0.67–0.94)**	**0.007**	**0.78 (0.65–0.92)**	**0.003**	184/2,969	**0.70 (0.57–0.85)**	**<0.001**	**0.71 (0.58–0.87)**	**0.001**
Total cholesterol	692/6,388					519/7,036				
- Poor	147/816	1.00		1.00		83/922	1.00		1.00	
- Intermediate	278/2,088	**0.73 (0.60–0.89)**	**0.002**	**0.74 (0.60–0.90)**	**0.003**	185/2,390	**0.76 (0.63–0.91)**	**0.003**	**0.77 (0.64–0.92)**	**0.004**
- Ideal	267/3,484	**0.49 (0.40–0.60)**	**<0.001**	**0.50 (0.41–0.61)**	**<0.001**	251/3,724	**0.50 (0.42–0.61)**	**<0.001**	**0.52 (0.43–0.63)**	**<0.001**
Blood pressure	692/6,388					519/7,036				
- Poor	230/967	1.00		1.00		200/1,200	1.00		1.00	
- Intermediate	287/2,176	**0.71 (0.60–0.85)**	**<0.001**	**0.72 (0.40–0.86)**	**<0.001**	200/2,429	**0.78 (0.64–0.95)**	**0.014**	**0.83 (0.68–1.01)**	**0.059**
- Ideal	175/3,245	**0.43 (0.35–0.53)**	**<0.001**	**0.44 (0.36–0.54)**	**<0.001**	119/3,407	**0.72 (0.57–0.92)**	**0.006**	**0.78 (0.61–0.99)**	**0.039**
Fasting blood glucose	692/6,388					519/7,036				
- Poor	147/535	1.00		1.00		130/676	1.00		1.00	
- Intermediate	158/1,008	**0.55 (0.44–0.69)**	**<0.001**	**0.56 (0.45–0.71)**	**<0.001**	135/1,175	**0.52 (0.42–0.63)**	**<0.001**	**0.51 (0.42–0.63)**	**<0.001**
- Ideal	387/4,845	**0.38 (0.31–0.46)**	**<0.001**	**0.40 (0.33–0.48)**	**<0.001**	254/5,185	**0.39 (0.32–0.46)**	**<0.001**	**0.41 (0.35–0.49)**	**<0.001**

CVH, Cardiovascular health; CVD, Cardiovascular disease; n/N, Number of CVD events/number of subjects by level of each metric. The hazard ratio (HR) and 95% CIs of each metric were estimated in separate Cox proportional hazard regression model. Model 1: Adjusted for sex and age. Model 2: Further adjusted for educational level, marital status, family history of CVD, and prevalent CVD (for all-cause mortality). The statistically significant data is bold.

Regarding mortality events, in a fully adjusted model, generally, both intermediate and ideal statuses of smoking, total cholesterol, BP, and FPG were associated with lower risk; however, only the intermediate status of BMI (0.80, 0.68–0.95), and the ideal status of physical activity (0.71, 0.58–0.87) were associated with the lower risk.

**FIGURE 1:**
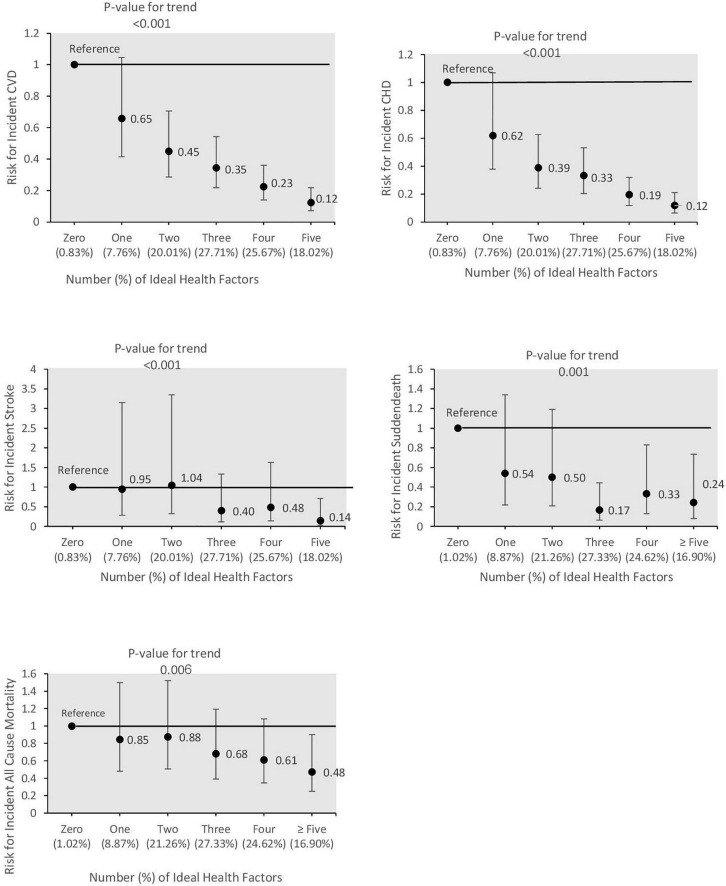
Hazard ratios (95% CI) of cardiovascular disease and its subtypes including coronary heart disease (CHD), stroke and sudden death, and all-cause mortality events according to combined ideal cardiovascular health metrics; Tehran Lipid and Glucose Study, 2005–20018. Hazard ratios adjusted for sex and age, educational level, marital status, family history of CVD, and prevalent CVD (for all-cause mortality).

HRs of global, behavioral, and biological ideal CVH per additional metrics for CVD, its different subtypes, and all-cause mortality are reported in Table 3. For CVD, CHD, and stroke, each additional metric of global, behavioral, and biological CVH significantly decreased the risk in both models 1 and 2. The risk of sudden death is only reduced with an increasing number of global and behavioral but not biological metrics in both models 1 and 2. For all-cause mortality, each additional metric of global, behavioral, and biological CVH decreased the risk by 13 (0.81–0.93), 18 (0.73–0.92), and 12% (0.80–0.98), respectively (model 2). Moreover, as shown in Figure 1, having a higher number of ideal CVH metrics was associated with a lower risk of CVD, CHD, stroke, sudden death, and all-cause mortality events (all P for trends < 0.05) and those getting ≥ 5 ideal CVH metrics had 88, 88, 86, 76, and 52% lower risk for these events, respectively, compared to those with zero ideal CVH metrics adjusting for confounders in model 2.

**TABLE 3:** Cox proportional hazard model for different cardiovascular events, and all-cause mortality (per one additional metric): Tehran Lipid and Glucose Study (TLGS), 2005–2018.

	Model 1	Model 2
	HR (95%CI)	HR (95%CI)
**Cardiovascular disease**		
Global cardiovascular health	**0.68 (0.64–0.73)**	**0.69 (0.65–0.73)**
Behavioral cardiovascular health	**0.73 (0.66–0.80)**	**0.73 (0.66–0.81)**
Biological cardiovascular health	**0.62 (0.57–0.67)**	**0.63 (0.57–0.68)**
**Coronary heart disease**		
Global cardiovascular health	**0.68 (0.63–0.72)**	**0.68 (0.64–0.73)**
Behavioral cardiovascular health	**0.71 (0.63–0.79)**	**0.71 (0.64–0.80)**
Biological cardiovascular health	**0.61 (0.56–0.67)**	**0.62 (0.56–0.68)**
**Stroke**		
Global cardiovascular health	**0.69 (0.59–0.80)**	**0.69 (0.60–0.80)**
Behavioral cardiovascular health	**0.77 (0.61–0.98)**	**0.78 (0.61–0.98)**
Biological cardiovascular health	**0.58 (0.47–0.72)**	**0.59 (0.48–0.73)**
**Sudden death**		
Global cardiovascular health	**0.72 (0.62–0.84)**	**0.75 (0.64–0.88)**
Behavioral cardiovascular health	**0.56 (0.43–0.72)**	**0.58 (0.45–0.76)**
Biological cardiovascular health	0.81 (0.65–1.00)	0.86 (0.69–1.06)
**All-Cause Mortality**		
Global cardiovascular health	**0.84 (0.78–0.90)**	**0.87 (0.81–0.93)**
Behavioral cardiovascular health	**0.79 (0.70–0.89)**	**0.82 (0.73–0.92)**
Biological cardiovascular health	**0.85 (0.77–0.94)**	**0.88 (0.80–0.98)**

Model 1: Adjusted for sex and age. Model 2: Further adjusted for educational level, marital status, family history of CVD, and prevalent CVD (for all-cause mortality). CVD, Cardiovascular disease. The statistically significant data is bold.

Multivariable HRs and 95% CI of association between global CVH categories and CVD are shown in Table 4. Accordingly, intermediate and ideal categories of CVH metrics were associated with a lower risk of CVD events in all of the models; the corresponding hazards were 0.56 (0.48–0.65) and 0.24 (0.17–0.35) in model 2, respectively. Moreover, age, having less than 6 years of education, being men, and family history of CVD significantly increased the risk of CVD events.

**TABLE 4:** Multivariable hazard ratios (HRs) and 95% confidence intervals (CI) for the association between global cardiovascular health categories * and cardiovascular disease: Tehran Lipid and Glucose Study (TLGS), 2005–2018.

	CVD	
	Model 1	Model 2
	HR (95%CI)	HR (95%CI)
Global Cardiovascular health*		
- Poor	1.00	1.00
- Intermediate	**0.55 (0.47–0.64)**	**0.56 (0.48–0.65)**
- Ideal	**0.23 (0.16–0.34)**	**0.24 (0.17–0.35)**
Age, year	**1.06 (1.06–1.07)**	**1.06 (1.05–1.07)**
Female (male as reference)	**0.61 (0.52–0.71)**	**0.55 (0.46–0.65)**
**Educational level**		
>12		**1.00**
6–12		**1.45 (1.10–1.92)**
<6		**1.38 (1.03–1.87)**
**Marital status**		
- Married		**1.00**
- Divorced + Widowed		**1.27 (1.01–1.60)**
Single		0.41 (0.17–1.00)
Family history of CVD, yes		**1.45 (1.22–1.72)**

CVD, Cardiovascular disease; T2DM, Type 2 diabetes mellitus.

*Defined according to the number of ideal metrics: 0–2 (poor), 3–4 (intermediate) and 5–6 (ideal). Model 1: Adjusted for sex and age. Model 2: Further adjusted for educational level, marital status, family history of CVD, and prevalent CVD (for all-cause mortality). The statistically significant data is bold.

Table 5 suggests multivariable HRs and 95% CI for the association between global CVH categories with subtypes of CVD and all-cause mortality. In model 2, intermediate and ideal categories of CVH metrics were significantly associated with 43 and 75% lower risk of CHD, 58 and 86% lower risk of stroke, 56 and 55% lower risk of sudden death, and 25 and 46% lower risk of all-cause mortality. When we deleted transient ischemic attack in the stroke outcome (*n* = 29) and re-did the data analysis for this event, the results were similar to those shown for total stroke events (i.e., the intermediate and ideal categories of CVH metrics were significantly associated with 57 (0.43, 0.29–0.65) and 86% (0.12, 0.03–0.51) lower risk of stroke in model 2, respectively).

**TABLE 5:** Multivariable hazard ratios (HR) and 95% confidence intervals (CI) for the association between global cardiovascular health categories *, CHD, stroke, sudden death and all- cause mortality: Tehran Lipid and Glucose Study (TLGS), 2005–2018.

	All-cause mortality	
	Model 1	Model 2
	HR (95%CI)	HR (95%CI)
**CHD**		
- Poor	**1**	**1**
- Intermediate	**0.56 (0.47–0.66)**	**0.57 (0.47–0.67)**
- Ideal	**0.24 (0.16–0.36)**	**0.25 (0.16–0.37)**
**Stroke**		
- Poor	**1**	**1**
- Intermediate	**0.41 (0.29–0.60)**	**0.42 (0.29–0.61)**
- Ideal	**0.13 (0.04–0.44)**	**0.14 (0.04–0.44)**
**Sudden death**		
- Poor	**1**	1
- Intermediate	**0.39 (0.26–0.60)**	**0.44 (0.29–0.66)**
- Ideal	**0.40 (0.18–0.89)**	**0.45 (0.21–0.99)**
**All-Cause Mortality**		
- Poor	1.00	1.00
- Intermediate	**0.69 (0.58–0.83)**	**0.75 (0.62–0.90)**
- Ideal	**0.49 (0.33–0.72)**	**0.54 (0.37–0.80)**

CVD, Cardiovascular disease; T2DM, Type 2 diabetes mellitus; CHD, coronary heart disease.

*Defined according to the number of ideal metrics: 0–2 (poor), 3–4 (intermediate), and 5–6 (ideal). Model 1: Adjusted for sex and age. Model 2: Further adjusted for educational level, marital status, family history of CVD, history of CVD, and prevalent CVD (for all-cause mortality). Model 3: Further adjusted for T2DM. The significant values are bold.

**FIGURE 2:**
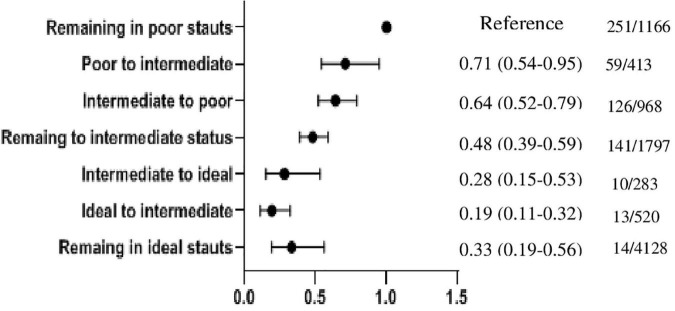
Hazard ratios (95% CI) of cardiovascular disease according to the change in ideal CVH from phase III (2005–2008) to phase IV (2008–2011). n/N: the number of CVD events/the number of total subjects in each category. Because of the limited number of CVD events in poor to ideal (*n* = 2) and ideal to poor (*n* = 0) categories, the HRs (95% CI) were not computed.

To show the robustness of our findings, we performed two sensitivity analyses. First, all of the above analysis was repeated in a subgroup of the TLGS population with dietary information (*n* = 1,755) among whom we found 163 (9.3%) and 57 (3.2%) CVD and all-cause mortality events, respectively. As shown in Supplementary Tables 1–6, the results were generally in line with our main findings, excluding the non-ideal nutritional status *per se* did not remain as a predictor for CVD events. Second, we assessed the impact of changes in the ideal CVH status from phase III (2005–2008) to phase IV (2008–2011) on CVD events in a subgroup analysis (5,666 out of 6,388 participants). Accordingly, participants were divided into nine categories as “remaining in poor status” (as reference categories), “poor to intermediate,” “poor to ideal,” “intermediate to poor,” “remaining in intermediate status,” “intermediate to ideal,” “ideal to poor,” “ideal to intermediate,” and “remaining in ideal status.” The association between the change in ideal CVH and the incident CVD event is shown in Figure 2. Compared with the reference category, the risk of CVD events for participants with staying in the intermediate and ideal statuses decreased by 52 (0.48, 0.39–0.59) and 67% (0.33, 0.19–0.56), respectively. The risk of CVD events for the participants with a favorable change in ideal CVH was reduced by 29 (0.71, 0.54–0.95) in the “poor to intermediate category” and 72% (0.28, 0.15–0.53) in the “intermediate to ideal category,” respectively. Among participants with an unfavorable change in ideal CVH, the risk of CVD events was reduced by 36 (0.64, 0.52–0.79) in the “intermediate to poor category” and 81% (0.19, 0.11–0.32) in the “ideal to intermediate category.” Because of the limited number of CVD events in poor to ideal (*n* = 2) and ideal to poor (*n* = 0) categories, the HRs (95% CI) were not computed.

## Discussion

Over more than a decade of follow-up in a population-based study conducted in the MENA region, we evaluated the association between CVH status with CVD and its subtypes, and all-cause mortality. According to our findings, each additional ideal CVH metric reduces the risk of CVD and its subtypes by at least a quarter. Additionally, we found that the intermediate and ideal CVH statuses lowered the risks of incident CVD by 44 and 76%, respectively, after adjustment for covariates including age, gender, education, marital status, and family history of CVD. Regarding all-cause mortality, the intermediate and ideal CVH statuses were associated with 25 and 46% lower risks, respectively.

It is difficult to compare cohort studies examining the relationship between ideal CVH status and CVD outcome due to differences in the definitions used for CVH status, level of confounder adjustment, mean age of study population, duration of follow-up, and various definitions for CVD outcomes. Despite this, in line with our study, a growing body of prospective studies and meta-analyses have demonstrated that a greater number of ideal CVH metrics is associated with a lower risk of CVD or all-cause mortality (7, 9–11, 24–26). Table 6 summarizes the studies in the field of ideal CVH and CVD outcomes that were performed after the meta-analysis conducted by Ramírez-Vélez et al. (7).

**TABLE 6:** Prospective investigations on associations between cardiovascular health metrics and risk of cardiovascular diseases and mortality.

First author, year (ref)	Country	Sample size	Age (years)	Men (%)	Follow-up duration	Main outcomes	Adjustments	Main findings
Zhou, 2018 (8)	China	938	35–59	49.6	20.3	CVD and all-cause mortality	Age, sex, urban or rural, northern or southern of China, types of work, education level, and drinking status	• Compared to those with 0–2 ideal CVH metrics, HRs (95% CIs) for CVD were 0.59 (95% CI 0.33–1.04) in the group with three and 0.24 (95% CI 0.12–0.47) in in the group with 4–7 ideal CVH metrics • Participants with 4–7 ideal CVH metrics had a 54% (95% CI 24–72%) lower risk of all-cause mortality in comparison with those with 0–2 ideal metrics.
Han, 2018 (9)	China	93,987	51.6 ± 12.0	40.2	15	CVD	Age, sex, living region, urbanization, drinking status, education level, family history of atherosclerotic CVD, and cohort sources	HRs (95% CIs) of CVD for those with 3, 4, 5, 6 and 7 ideal CVH metrics were 0.83 (0.74–0.93), 0.66 (0.59–0.74), 0.55 (0.48–0.61), 0.44 (0.38–0.50) and 0.24 (0.18–0.31) compared with participants having ≤ 2 ideal CVH metrics
Isiozor, 2019 (25)	Finland	2,584	40–62	100	25.2	AMI	Age, alcohol consumption, socioeconomic status, history of coronary heart disease, and history of type 2 diabetes mellitus	• ↑ Ideal CVH metrics →↓ risk AMI HR (95% CI) for AMI in the intermediate [0.58 (0.47–0.71)] and ideal [0.29 (0.15–0.57)] vs. poor categories of CVH metrics
Dong, 2019 (10)	China	8,754	35–64	63.6	6.3	CVD and all-cause mortality	Age, sex, education attainment, marriage status, and region	• ↑Ideal CVH metrics (categorical variable) →↓risk of CVD, all-cause mortality • HR (95% CI) for CVD [0.75 (0.67–0.85)] and all-cause mortality [0.78 (0.70–0.88)] per additional ideal metric
Yang, 2021 (11)	Korea	208, 673	≥ 75	42.5	10	CVD, all-cause mortality, and cause-specific mortality	Age, sex, economic status, hospital frailty score, living in metropolitan cities, and competing risk of death	• Ideal CVH metrics (categorical variable) →↓risk of CVD, all-cause mortality, CHD, ischemic stroke/systemic embolism in the both elderly and the very elderly participants • HR (95% CI) for CVD was 0.82 (0.81–0.83) and all-cause mortality was 0.93 (0.91–0.94) in the elderly participants per additional ideal metric • HR (95% CI) for CVD was 0.86 (0.84–0.88) and all-cause mortality was 0.95 (0.93–0.97) in the very elderly participants per additional ideal metric
Wang, 2021 (12)	China	180,515	30–70	81.2	4	CVD	Age	↓ Lifetime risk for CVD and specific-CVD of MI, and stroke by ↑ the number of ideal CVH metrics

The table included studies in the field of ideal CVH and CVD outcomes among the general population that were performed after the meta-analysis conducted by Ramírez-Vélez et al. (7). CI, confidence interval; CVD, cardiovascular disease; CVH, cardiovascular health, HR, hazard ratio; AMI, acute myocardial infarction.

According to a national trend in ideal CVH metrics in Iranian adults, among the whole population, the prevalence of individuals having 5, 6, or 7 CVH metrics during 2007–2016 decreased, although the mean CVH metrics during this period increased from 4.7 to 5.0 (14). The high prevalence of obesity, hypertension, T2DM, and hypercholesterolemia was reported among residents in the MENA region (13). Moreover, we showed that in the city of Tehran, about 60% of the population attributable fraction (PAF) of CVD events were related to hypercholesterolemia, hypertension, T2DM, and current smoking (27). Hence, the finding that a one unit increase in ideal CVH metric among the Iranian population was associated with a 31% lower risk of CVD and a 13% lower risk of all-cause mortality indicated that there were incremental benefits in terms of both CVD and mortality events with the increasing number of ideal CVH metrics. Similarly, in the CVD-China study, one unit increase in the CVH metric was associated with a 25 and 22% lower risk of CVD and all-cause mortality events (10). Another study on the Chinese population found that increasing the ideal CVH score by one point reduced the risk of CVD events by 18% in the full adjusted model (9). The Korea National Health Insurance Service senior cohort conducted among the elderly population reported a decreased risk of CVD and all-cause mortality events with an increasing number of CVH metrics (11). A meta-analysis including six prospective cohorts showed that each increase in ideal CVH was associated with a lower risk of 19 and 11% for CVD and all-cause mortality events, respectively (26).

In this study, we also found that having intermediate and ideal categories of ideal CVH was generally associated with a lower risk of CVD events and all-cause mortality events. The meta-analysis, which included over 210,000 adults, discovered that those with intermediate and ideal categories had 47 and 72% lower risks for incident CVD events, respectively, compared to high-risk individuals (7). Another meta-analysis by Guo et al. also showed that comparing the most to the least category of ideal CVH, the relative risk for CVD and all-cause mortality was reduced by 78 and 70%, respectively (26). In a subgroup analysis, we also found that compared to those remaining in the poor category, all of the categories had a lower risk for CVD events. Moreover the “intermediate to ideal category” had a 72% lower risk for CVD events. Our findings are in line with those of population-based cohort studies that show favorable changes in ideal CVH reduced risk of chronic diseases such as arterial stiffness and type 2 diabetes (28, 29).

There was a well-known but not a consistent association between components of ideal CVH metrics, including biological and behavioral factors and CVD and all-cause mortality events. In the Guo et al. meta-analysis among 7 CVH metrics, BMI and healthy diet score did not have a strong association with cardiovascular and all-cause mortality events (26). In the current study, ideal nutritional status was not associated with CVD events in our subgroup analyses. This may be because adherence to a healthy diet was low with a median (IQR) score of 2 (2, 3) and more than 70% of our population was categorized as intermediate. Therefore, low variation between individuals may account for the lack of association between the diet metric and ideal CVH in this subgroup analysis. In addition, the lack of observed association between four out of five components of diet metric, which include whole grains, fruit and vegetables, fish, and sodium, may be due to the insufficient power (0.07–0.71) to detect such association. Regarding SSBs with a power of 0.89, more than 96% of subjects were categorized as ideal. In our previous studies, we found that consumption of SSBs of more than 7 ounces/week increased the risk of chronic diseases such as metabolic syndrome and obesity (30, 31); suggesting that classification of SSBs based on < 36 ounces/week might not be appropriate for our population. Furthermore, all the ideal categories of CVH metrics, except for BMI were associated with a lower risk of all-cause mortality. A standardized case-control study of acute myocardial infarction in 52 countries by Yusuf et al. found that abnormal lipids, smoking, alcohol consumption, hypertension, diabetes, abdominal obesity, lower consumption of fruits, vegetables, and physical inactivity account for 90% of the population at risk in both genders (32). Similarly, among the Iranian population, traditional risk factors contribute to about 89% and 74% of premature CVD events (33). Our previous studies in the city of Tehran as well as national studies in the country revealed that the prevalence of ideal CVH is very small (14, 34). Hence, in line with our data analysis and as acknowledged by Ramírez-Vélez et al., a more achievable goal, particularly in developing countries such as Iran, might be to meet at least 3–4 ideal CVH metrics that are significantly associated with a lower risk of CVD (7). In our data analysis, excluding the prevalence of ideal smoking status (79.4%), the prevalence of other ideal CVH metrics was not favorable. For example, only about one-quarter had a normal BMI, and only about half had ideal BP and cholesterol levels. This finding suggests immediate community-based lifestyle intervention by health policymakers to promote a healthy lifestyle, increase physical activity, and restrict salt intake should be planned (35). Actually, we recently showed that community-based lifestyle educational intervention in Tehran reduced the incidence of metabolic syndrome by 20% over 6 years, with the favorable impact mainly derived from improvement in lipid profile and glucose level (36).

## Strengths and limitations

This study has numerous strengths, including its prospective study design, large sample size, precise measurements rather than relying on self-reported ideal CVH metrics, potential confounders, and adjudication of CVD events and mortality outcomes. This is also the first study in the MENA region to assess the relationship between ideal CVH and CVD events and all-cause mortality outcomes.

There are also some limitations to our study. First, TLGS uses validated methods of CVD event ascertainment, but it cannot fully account for events that are not present clinically (e.g., silent myocardial infarction). Second, the study was conducted in the metropolitan city of Tehran, therefore our findings might not extrapolate to the whole country, especially the rural zones. Lastly, according to TLGS design, the data including duration for quitting smoking, sleep health, and A1C are not available to calculate ideal CVH based on updated metrics for measurement and quantitative assessment of CVH (37).

## Conclusion

During more than a decade of follow-up in a middle-aged Iranian population, we found that all of the individual ideal CVH metrics were strong predictors, except for intermediate physical activity level for CVD, BMI < 25 kg/m^2^, and intermediate physical activity for all-cause mortality. However, we found that meeting a greater number of ideal CVH metrics reduces CVD, CHD, stroke, sudden death, and all-cause mortality. So, following better lifestyle habits and improving ideal CVH might potentially reduce the burden of CVD events and mortality outcomes in our country; the issue needs immediate population-based trials.

## Data availability statement

The raw data supporting the conclusions of this article will be made available by the authors, without undue reservation.

## Ethics statement

The studies involving human participants were reviewed and approved by Shahid Beheshti University of Medical Sciences Ethics Committee. The patients/participants provided their written informed consent to participate in this study.

## Author contributions

FH, ND, and HT drafted the manuscript. SH-N, MH, and NM analyzed the data. FH and FA critically revised the manuscript. FH supervised the team. All authors contributed to the article and approved the submitted version.
