# Supplementary material for: Ideal cardiovascular health status and risk of cardiovascular disease and all-cause mortality: over a decade of follow-up in the Tehran lipid and glucose study
Source: Front Cardiovasc Med. 2022 Aug 4;9:898681. doi: 10.3389/fcvm.2022.898681 (PMC9386047; doi:10.3389/fcvm.2022.898681)
Supplement: Supplementary file 1 [file Data_Sheet_1.pdf]

Supplementary Table 1. Baseline Characteristics by Global Cardiovascular Health Status

		Global Cardiovascular Health Status*			
	**Overall (n=1755)**	**Poor (n=449)**	**Intermediate (n=929)**	**Ideal (n=377)**	***P*-value**
**Continuous variables, Mean ± SD**					
**Age (year)**	45.6±10.8	49.5±10.7	45.6±10.6	41.1±9.8	**< 0.001**
**BMI (kg/m²)**	28.0±4.6	29.9±4.1	28.2±4.3	25.2±4.2	**< 0.001**
**SBP (mmHg)**	114.3±17.4	125.0±15.6	113.6±17.2	103.3±11.7	**< 0.001**
**DBP (mmHg)**	75.2±10.5	81.6±9.4	74.8±10.2	68.4±7.4	**< 0.001**
**FPG (mg/dL)**	88.0 (82.0-95.0)	96.0 (88.0-111.0)	88.0 (82.0-93.0)	84.0 (79.5-89.0)	**< 0.001**
**Total cholesterol (mg/dL)**	195.4±37.9	216.1±36.4	195.3±37.1	170.9±25.1	**< 0.001**
**HDL cholesterol (mg/dL)**	42.1±10.2	39.8±9.6	41.9±10.0	44.9±10.5	**< 0.001**
**Categorical variables, number (%)**					
**Sex (male)**	788(44.9)	260(57.9)	420(45.2)	108(28.6)	**< 0.001**
**Educational level**					
**≤6**	372(21.2)	138(30.7)	190(20.5)	44(11.7)	**< 0.001**
**6-12**	986(56.2)	225(50.1)	529(56.9)	232(61.5)	**< 0.001**
**>12**	397(22.9)	86(19.2)	210(22.6)	101(26.8)	**< 0.001**
**Low Physical activity**	639 (36.4)	241 (53.7)	338 (36.4)	60 (15.9)	**< 0.001**
**Current smoking,**	233 (13.3)	98 (21.8)	115 (12.4)	20 (5.3)	**<0.001**
**Marital status**					
**Married**	1542(87.9)	392(87.3)	814(87.6)	336(89.1)	**< 0.001**
**Widowed + Divorced**	116(6.6)	44(9.8)	58(6.2)	14(3.7)	**< 0.001**
**Single**	97(5.5)	13(2.9)	57(6.1)	27(7.2)	**< 0.001**
**Glucose lowering drug use, yes**	82 (4.7)	52(11.6)	27(2.9)	3(0.8)	**< 0.001**
**Anti-hypertensive drug use, yes**	76(4.3)	39(8.7)	34(3.7)	3(0.8)	**< 0.001**
**Lipid-lowering drug use, yes**	73(4.2)	44(9.8)	29(3.1)	0(0)	**< 0.001**
**Family history of CVD, yes**	393(77.6)	105(23.4)	197(21.2)	91(24.1)	0.434
**T2DM**	133(7.6)	87(19.4)	43(4.6)	3(0.8)	**< 0.001**

Values are mean ± SD or n (%). *Defined according to the number of ideal metrics: 0 to 2 (poor), 3 to 4 (intermediate), and 5 to 7 (ideal). BMI, Body mass index; SBP, Systolic blood pressure; DBP, diastolic blood pressure; FPG, Fasting plasma glucose; T2DM, Type 2 diabetes mellitus

Supplementary Table 2. Hazard ratios of intermediate and ideal CVH for Cardiovascular disease: Analysis by Metric

	n/N	Model 1		Model 2	
		HR (95%CI)	P-value	HR (95%CI)	P-value
**Smoking status**	163/1755				
- **Poor**	30/233	1.00		1.00	
- **Intermediate**	20/161	0.69 (0.39-1.23)	0.214	0.72 (0.40-1.25)	0.712
**Ideal**	113/1361	0.73 (0.48-1.12)	0.736	0.75 (0.48-1.15)	0.750
**Body mass index**	163/1755				
- **Poor**	59/529	1.00		1.00	
- **Intermediate**	70/769	**0.59 (0.41-0.85)**	**0.004**	**0.62 (0.43-0.89)**	**0.011**
**Ideal**	34/457	**0.37 (0.23-0.58)**	**0.373**	**0.39 (0.24-0.61)**	**<0.001**
**Physical activity**	163/1755				
- **Poor**	59/639	1.00		1.00	
- **Intermediate**	40/399	1.03 (0.68-1.54)	0.883	1.02 (0.68-1.53)	0.915
**Ideal**	64/717	0.96 (0.67-1.37)	0.832	0.92 (0.64-1.31)	0.648
**Total cholesterol**	163/1755				
- **Poor**	37/215	1.00		1.00	
- **Intermediate**	66/569	**0.65 (0.43-0.98)**	**0.043**	**0.66 (0.44-0.99)**	**0.049**
**Ideal**	60/971	**0.41 (0.27-0.63)**	**<0.001**	**0.42 (0.27-0.64)**	**<0.001**
**Blood pressure**	163/1755				
- **Poor**	48/228	1.00		1.00	
- **Intermediate**	70/601	0.78 (0.54-1.13)	0.196	0.79 (0.54-1.15)	0.222
**Ideal**	45/926	**0.48 (0.31-0.74)**	**0.001**	**0.48 (0.31-0.75)**	**0.001**
**Fasting blood glucose**	163/1755				
- **Poor**	22/110	1.00		1.00	
- **Intermediate**	34/221	0.83 (0.48-1.43)	0.515	0.91 (0.52-1.56)	0.908
**Ideal**	107/1424	**0.52 (0.33-0.83)**	**0.006**	**0.55 (0.35-0.89)**	**0.014**
**Healthy diet**	163/1755				
- **Poor**	25/402	1.00		1.00	
- **Intermediate**	134/1258	1.26 (0.82-1.95)	0.279	1.30 (0.84-2.00)	0.234
**Ideal**	4/95	0.98 (0.48-1.98)	0.966	1.05 (0.52-2.14)	0.878

CVH, Cardiovascular health
n/N: Number of CVD events /number of subjects by level of each metric
The Hazard ratios and 95% CIs of each metric were estimated in separate Cox proportional hazard regression model
Model 1:Adjusted for sex and age
Model 2: Further adjusted for educational level, marital status, family history of CVD, and prevalent CVD (for all-cause mortality).

Supplementary Table 3. Hazard ratios (HRs) of cardiovascular disease for each components of healthy diet metric

	n/N	Model 1		Model 2	
		HRs (95%CI)	*P*-value	HR (95%CI)	*P*-value
**Whole grains**	163/1755				
**Poor**	97/1115	1			
**Ideal**	66/640	0.88 (0.64-1.21)	0.451	0.89 (0.65-1.23)	0.513
**Fruits and vegetables**	163/1755				
**Poor**	67/692	1		1	
**Ideal**	96/1063	0.81 (0.59-1.10)	0.190	0.83 (0.60-1.13)	0.251
**Fish**	163/1755				
**Poor**	136/1472	1		1	
**Ideal**	27/283	0.91 (0.60-1.38)	0.188	0.94 (0.62-1.44)	0.801
**Sugar sweetened beverages**	163/1755				
**Poor**	3/61	1		1	
**Ideal**	160/1694	1.35 (0.43-4.28)	0.600	1.46 (0.46-4.62)	0.516
**Sodium**	163/1755				
**Poor**	155/1690	1		1	
**Ideal**	8/65	1.22 (0.60-2.48)	0.580	1.23 (0.60-2.52)	0.555

Ideal defined as at least 4.5 cups/day of fruits and vegetables; at least two 3.5-ounce servings/week of fish; <1500 mg/day of sodium; ≤ 36 ounces/week of sugar sweetened beverages (SSBs); and at least three 1-ounce servings/ day of whole grains
n/N: Number of CVD events /number of subjects by level of each metric
The Hazard ratios and 95% CIs of each metric were estimated in separate Cox proportional hazard regression model
Model 1: Adjusted for sex and age
Model 2: Further adjusted for educational level, marital status, family history of CVD, and prevalent CVD (for all-cause mortality).

Supplementary Table 4. Cox proportional hazard for incident cardiovascular disease and all-cause mortality (per one additional metric ideal CVH)

	Cardiovascular Disease		All-cause Mortality	
	**Model 1**	**Model 2**	**Model 1**	**Model 2**
**Global cardiovascular health**	**0.72 (0.64-0.81)***	**0.72 (0.64-0.81)**	**0.75 (0.61-0.93)**	**0.76 (0.62-0.94)**
**Behavioral cardiovascular health**	**0.78 (0.65-0.94)**	**0.78 (0.64-0.94)**	0.74 (0.54-1.02)	0.75 (0.55-1.03)
**Biological cardiovascular health**	**0.62 (0.52-0.74)**	**0.63 (0.52-0.75)**	**0.72 (0.53-0.97)**	**0.73 (0.54-0.99)**

CVH, Cardiovascular health
***Hazard ratios (95% confidence interval)**
Model 1: Adjusted for sex and age
Model 2: Further adjusted for educational level, marital status, family history of CVD, and prevalent CVD (for all-cause mortality).

Supplementary Table 5. Multivariable hazard ratios (95% CI) of association between global cardiovascular health categories and cardiovascular disease

	Cardiovascular disease	
	Model 1	Model 2
**Global Cardiovascular health***		
- **Poor**	1.00	1.00
- **Intermediate**	**0.56 (0.40-0.76)**	**0.56 (0.41-0.77)**
- **Ideal**	**0.28 (0.15-0.52)**	**0.28 (0.16-0.53)**
**Age, year**	**1.08 (1.06-1.09)**	**1.07 (1.06-1.09)**
**Female (male as reference)**	**0.50 (0.36-0.71)**	**0.48 (0.34-0.69)**
**Educational level**		
- **>12**		**1.00**
- **6-12**		**1.15 (0.72-1.84)**
- **<6**		**1.51 (0.91-2.51)**
**Marital status**		
- **Married**		**1.00**
- **Divorced + Widowed**		**1.01 (0.57-1.76)**
- **Single**		0.26 (0.04-1.92)
**Family history of CVD, yes**		**1.31 (0.92-1.84)**
**T2DM, yes**		

CVD: Cardiovascular disease; T2DM: Type 2 diabetes mellitus
***Defined according to the number of ideal metrics: 0 to 2 (poor), 3 to 4 (intermediate) and 5 to 6 (ideal).**
Model 1: Adjusted for sex and age
Model 2: Further adjusted for educational level, marital status, family history of CVD, and prevalent CVD (for all-cause mortality)

Supplementary Table 6. Multivariable hazard ratios (95% CI) of association between global cardiovascular health categories and All- cause mortality

	All-cause mortality	
	**Model 1**	**Model 2**
**Global cardiovascular health***		
- **Poor**	1.00	1.00
- **Intermediate**	0.61 (0.36-1.06)	0.64 (0.37-1.11)
- **Ideal**	**0.31 (0.11-0.89)**	**0.32 (0.11-0.93)**
**Age, year**	**1.10 (1.08-1.12)**	**1.09 (1.06-1.12)**
**Female (male as reference)**	**0.42 (0.23-0.75)**	**0.36 (0.19-0.69)**
**Educational level**		
- **>12**		**1.00**
- **6-12**		0.90 (0.39-2.11)
- **<6**		1.80 (0.77-4.18)
**Marital status**		
- **Married**		1.00
- **Divorced + Widowed**		0.89 (0.34-2.29)
- **Single**		1.31 (0.17-9.93)
**Family history of CVD, yes**		0.94 (0.49-1.79)
**T2DM, yes**		

CVD: Cardiovascular disease; T2DM: Type 2 diabetes mellitus
***Defined according to the number of ideal metrics: 0 to 2 (poor), 3 to 4 (intermediate) and 5 to 6 (ideal)**
Model 1: Adjusted for sex and age
Model 2: Further adjusted for educational level, marital status, family history of CVD, history of CVD, and prevalent CVD (for all-cause mortality)
